# Caffeylpyruvate hydrolase from the bioluminescent fungus *Neonothopanus gardneri* is the key recycling enzyme in the fungal bioluminescence pathway

**DOI:** 10.1111/febs.70554

**Published:** 2026-05-19

**Authors:** Caio K. Zamuner, Douglas M. M. Soares, Bianca B. Nóbrega, Etelvino J. H. Bechara, Zinaida M. Kaskova, Alexander S. Mishin, Karen S. Sarkisyan, Ilia V. Yampolsky, Cassius V. Stevani

**Affiliations:** ^1^ Departamento de Química Fundamental, Instituto de Química Universidade de São Paulo Brazil; ^2^ Departamento de Bioquímica, Instituto de Química Universidade de São Paulo Brazil; ^3^ Department of Bioprocess Engineering and Biotechnology, School of Pharmaceutical Sciences São Paulo State University Araraquara Brazil; ^4^ Shemyakin‐Ovchinnikov Institute of Bioorganic Chemistry Russian Academy of Sciences Moscow Russia; ^5^ Pirogov Russian National Research Medical University Moscow Russia; ^6^ Planta LLC Moscow Russia; ^7^ Synthetic Biology Group MRC Laboratory of Medical Sciences London UK; ^8^ Institute of Clinical Sciences Faculty of Medicine, and Imperial College Centre for Engineering Biology, Imperial College London London UK; ^9^ Light Bio Inc. Ketchum ID USA

**Keywords:** basidiomycete, fumarylacetoacetate hydrolase superfamily, fungal bioluminescence, retro aldol condensation

## Abstract

Recent studies uncovered the fungal bioluminescence pathway (FBP), a biosynthetic signaling axis to which caffeic acid is central. A key step, the hydrolysis of oxyluciferin into caffeic and pyruvic acids, was previously observed only in crude extracts, with pyruvate formation uncertain. Using gene homology, we identified and cloned *cph* from *Neonothopanus gardneri*, encoding a predicted fumarylacetoacetate hydrolase (FAH)‐family enzyme, named ngarCPH. We successfully expressed and purified ngarCPH and demonstrated its catalytic activity on oxyluciferin, confirming formation of both products. Kinetic characterization revealed the optimal temperature and pH, while structural modeling and multiple sequence alignment indicate characteristics similar to those of FAH enzymes. This work not only expands the knowledge of the function of enzymes from the FAH superfamily but also confirms a complete, self‐sustained bioluminescence cycle in fungi. Our findings support the future development of autonomous bioluminescent systems in heterologous hosts.

## Graphical abstract

Caffeic acid is a central metabolite in the fungal bioluminescence pathway. We identified and characterized caffeylpyruvate hydrolase from *Neonothopanus gardneri* (ngarCPH) and demonstrate its ability to hydrolyze fungal oxyluciferin into caffeic and pyruvic acids, confirming a complete and self‐sustained fungal bioluminescence cycle.

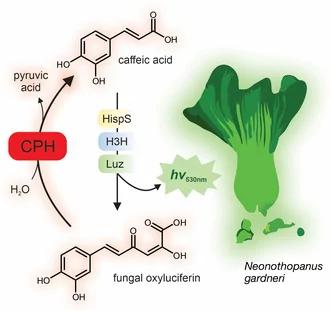

**Abbreviations**
Abs_380nm_
: Absorption at 380 nm
agCPH: caffeylpyruvate hydrolase from *Armillaria gallica*
aamCPH: caffeylpyruvate hydrolase from *Armillaria mellea*
ANOVA: Analysis of variance
AoCPH: caffeylpyruvate hydrolase from *Armillaria ostoyae*
Asp: Aspartate
C3: Three‐carbon
cDNA: complementary DNA
cg1458: Oxaloacetate decarboxylase from *Corynebacterium glutamicum*
cgFPH1: Fumarylpyruvate hydrolase from *Corynebacterium glutamicum*
CPH: Caffeylpyruvate hydrolase
*cph*
: caffeylpyruvate hydrolase gene
DNA: Deoxyribonucleic acid
eaFAH: Fumarylacetoacetate hydrolase from *Exiguobacterium antarcticum*
ESI: Electrospray ionization
FAH: Fumarylacetoacetate hydrolase
FBP: Fungal Bioluminescence Pathway
FPH‐A: Fumarylacetoacetate hydrolase subfamily A
Glu: Glutamate
H3H: Hispidin‐3‐hydroxylase
HEI: High‐energy intermediate
His: Histidine
HispS: Hispidin synthase
HPLC: High‐performance liquid chromatography
IPTG: Isopropyl β‐D‐1‐thiogalactopyranoside
k_cat_
: Turnover number
K_M_
: Michaelis constant
LB: Luria–Bertani
LRA6: L‐2,4‐diketo‐3‐deoxyrhamnonate hydrolase from *Sphingomonas sp*
Luz: Fungal luciferase
mcCPH: caffeylpyruvate hydrolase from *Mycena chlorophos*
mcH3H: Hispidin‐3‐hydrolase from *Mycena chlorophos*
mkCPH: caffeylpyruvate hydrolase from *Mycena kentingensis*
MS: Mass spectrometry
msCPH: caffeylpyruvate hydrolase from *Mycena sanguinolenta*
mvCPH: caffeylpyruvate hydrolase from *Mycena venus*
MW: Molecular weight
ngarCPH: caffeylpyruvate hydrolase from *Neonothopanus gardneri*
Ni‐NTA: Nickel–nitrilotriacetic acid
nnCPH: caffeylpyruvate hydrolase from *Neonothopanus nambi*
nnLuz: Fungal luciferase from *Neonothopanus nambi*
NRPS‐PKS: Nonribosomal peptide synthetase‐polyketide synthase
OD_600nm_
: Optical density at 600 nm
PA: Analytical grade
PAE: Predicted Aligned Error
paFPH2: Fumarylpyruvate hydrolase from *Pseudomonas aeruginosa*
PCR: Polymerase Chain Reaction
PDA: Photodiode array detector
pLDDT: predicted Local Distance Difference Test
PMSF: Phenylmethylsulfonyl fluoride
RLU: Relative light units
RNA: Ribonucleic acid
R_t_
: Retention time
RT: Room temperature
ssMPH: Maleylpyruvate hydrolase from *Sphingobium* sp. SYK‐6
UV–Vis: Ultraviolet–visible
V_max_
: Maximum velocity
ε_380nm,oxy_
: Molar absorption coefficient of fungal oxyluciferin at 380 nm
λ_em_
: Emission wavelength
λ_ex_
: Excitation wavelength

## Introduction

The light emission seen in bioluminescent fungi is the result of the oxidation of fungal luciferin, a compound biosynthesized by consecutive biochemical steps in the transformation of caffeic acid [1]. This biological process involves four key enzymes and is commonly referred to as the fungal bioluminescence pathway (FBP) [2] (Fig. 1A). Hispidin synthase (HispS), a hybrid fungal nonribosomal peptide synthetase‐polyketide synthase (NRPS‐PKS) [3], catalyzes the addition of carbon units to caffeic acid to produce the styrylpyrone hispidin. Hispidin‐3‐hydroxylase (H3H) catalyzes the monooxygenation of hispidin at C3 of the α‐pyrone ring, yielding 3‐hydroxyhispidin, the fungal luciferin. The light‐emitting reaction involves the luciferase (Luz), which oxidizes the fungal luciferin by inserting molecular oxygen, forming the high‐energy intermediate (HEI) endoperoxide. During the decomposition of HEI into the oxyluciferin (caffeylpyruvate acid) photons are released, determining the intrinsic feature of emission maximum wavelength as 530 nm in bioluminescent fungi [1].

**Fig. 1:**
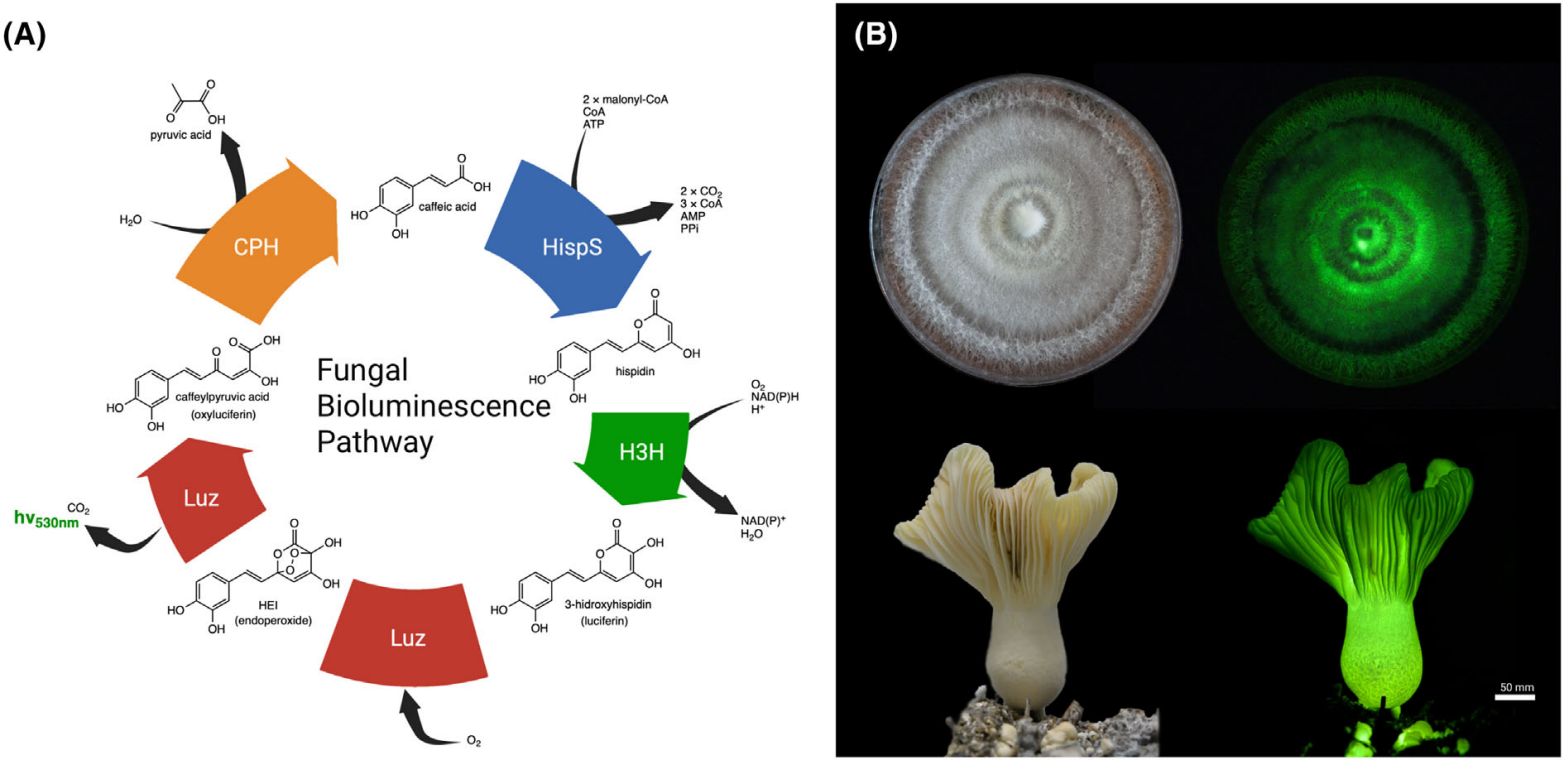
Fungal bioluminescence pathway (FBP). (A) The biochemical pathway that accounts for the bioluminescence in basidiomycetes involves four enzymes: HispS, H3H, Luz, and CPH which are, respectively, hispidin synthase, hispidin‐3‐hydroxylase, luciferase, and caffeylpyruvate hydrolase. (B) Bioluminescence in the *N. gardneri* mycelium in a 100‐mm Petri dish (top) and mushroom (bottom).

The final stage of the FBP, the recycling of oxyluciferin into caffeic acid, was proposed to be enzymatically catalyzed by a soluble protein named caffeylpyruvate hydrolase (CPH). Some evidence supports this hypothesis, including an increased signal for caffeic acid in reactions containing oxyluciferin and partially purified protein extracts from the bioluminescent mycelia of *Neonothopanus gardneri* (Fig. 1B) [4], as well as the location of a gene encoding a conserved domain for enzymes involved in the hydrolysis of aromatic compounds near the FBP cluster in the genome of some bioluminescent fungi [2, 5]. Therefore, preliminary results suggest the hypothesis of a functional role for CPH in fungal bioluminescence, which still needs to be confirmed.

Since the enzymes of the FBP perform conserved functions across bioluminescent fungi [5, 6], validating the catalytic role of CPH offers valuable complementary insights into this newly characterized metabolic pathway. This is especially relevant for understanding why a step not strictly required for light emission has nonetheless been evolutionarily maintained. Moreover, the fungal FBP has emerged as an important tool for bioimaging, particularly in plants [7, 8, 9]. Plants possess endogenous metabolic pathways for caffeic acid biosynthesis, which can be redirected, through genetic engineering of the FBP, toward luciferin production, enabling sustained and autonomous bioluminescence. However, it remains unclear whether the consumption of caffeic acid, normally allocated to lignin biosynthesis [10], may interfere with cellular homeostasis and impair long‐term plant development. Demonstrating that CPH exhibits the catalytic activity proposed in the literature [4] is essential to support its application as a mechanism to reduce intracellular caffeic acid levels through oxyluciferin recycling. Although the biological importance of CPH has not yet been fully understood, previous studies have shown that the inclusion of CPH in plants prolongs light emission [9], highlighting its potential role in minimizing metabolic interference.

In this study, we report the successful cloning of the coding sequence for the CPH enzyme from the bioluminescent fungus *N. gardneri* (ngarCPH), followed by its expression in a bacterial system and subsequent purification of recombinant protein for enzymatic assays studies. Caffeic and pyruvic acids were identified as products of the CPH‐catalyzed oxyluciferin hydrolysis reaction, confirming their functional role in the final step of the fungal bioluminescence pathway. CPH enzymes belong to the fumarylacetoacetate hydrolase (FAH) superfamily, and high‐confidence structural models obtained from AlphaFold predictions, together with multiple sequence alignments, suggest a carbon–carbon bond hydrolysis mechanism similar to that observed in those enzymes. These results corroborate the enzymatic nature of caffeic acid recycling in certain bioluminescent fungi and create new opportunities for future studies that explore its physiological function from an evolutionary perspective. At the same time, they contribute to the development of optimized heterologous light‐emitting systems.

## Results

### Cloning, recombinant expression, purification, and functional characterization of ngarCPH

The fungus *N. gardneri* is one of the largest and brightest bioluminescent fungal species described to date, found attached to the base of babassu palms (*Attalea speciosa*) in the Palm Forest—a transition zone in northeastern Brazil characterized by native palm species, such as the babassu and carnauba (*Copernicia prunifera*). Initially, the 897‐bp long *cph* coding sequence was successfully amplified from cDNA samples obtained from the reverse transcription of total RNA isolated from the fungal mycelium (Fig. S1 A). The *cph* coding sequence was cloned into pET‐28b vectors to express the recombinant C‐terminal His‐tagged ngarCPH in *Escherichia coli* cells. After sequencing the plasmid, an inconsistency was identified in some nucleotide sequences (BLASTn, 98.18% identity and 91% coverage) (Fig. 2A) and in the corresponding amino acid sequences after translation (BLASTp, 97.26% identity and 90% coverage) when compared to the previously deposited sequence [2] (Fig. 2B).

**Fig. 2:**
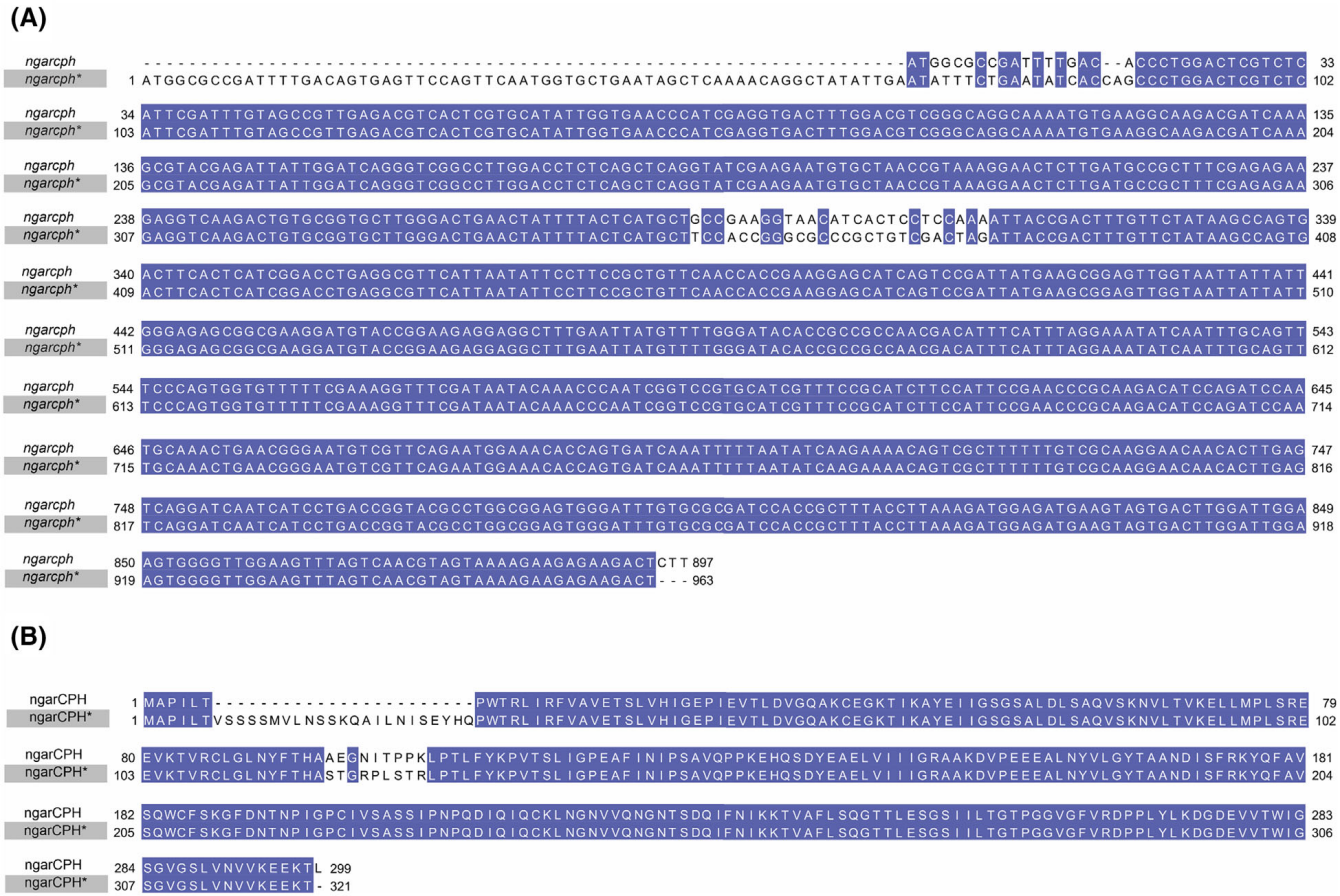
Alignment of coding and protein sequences of caffeylpyruvate hydrolase (CPH) from *N. gardneri*. (A) Nucleotide coding sequence, and (B) amino acid sequence of ngarCPH. Sequences assigned as ngarcph/ngarCPH are from this study, whereas ngarcph*/ngarCPH* are from literature [2]. Blue highlighting indicates consensus sequences. The stop codon is not shown.

Denaturing gel electrophoresis indicated that the recombinant ngarCPH has a molecular weight of approximately 33 kDa (Fig. S1 B) and is predominantly localized in the bacterial pellet (Fig. 3A). With this, even with low amounts of protein, approximately 1 mg per liter of culture, it was possible to recover ngarCPH after elution from the Ni‐NTA affinity column using an imidazole concentration gradient above 150 mm. The purity was confirmed by size exclusion chromatography (Fig. S2).

**Fig. 3:**
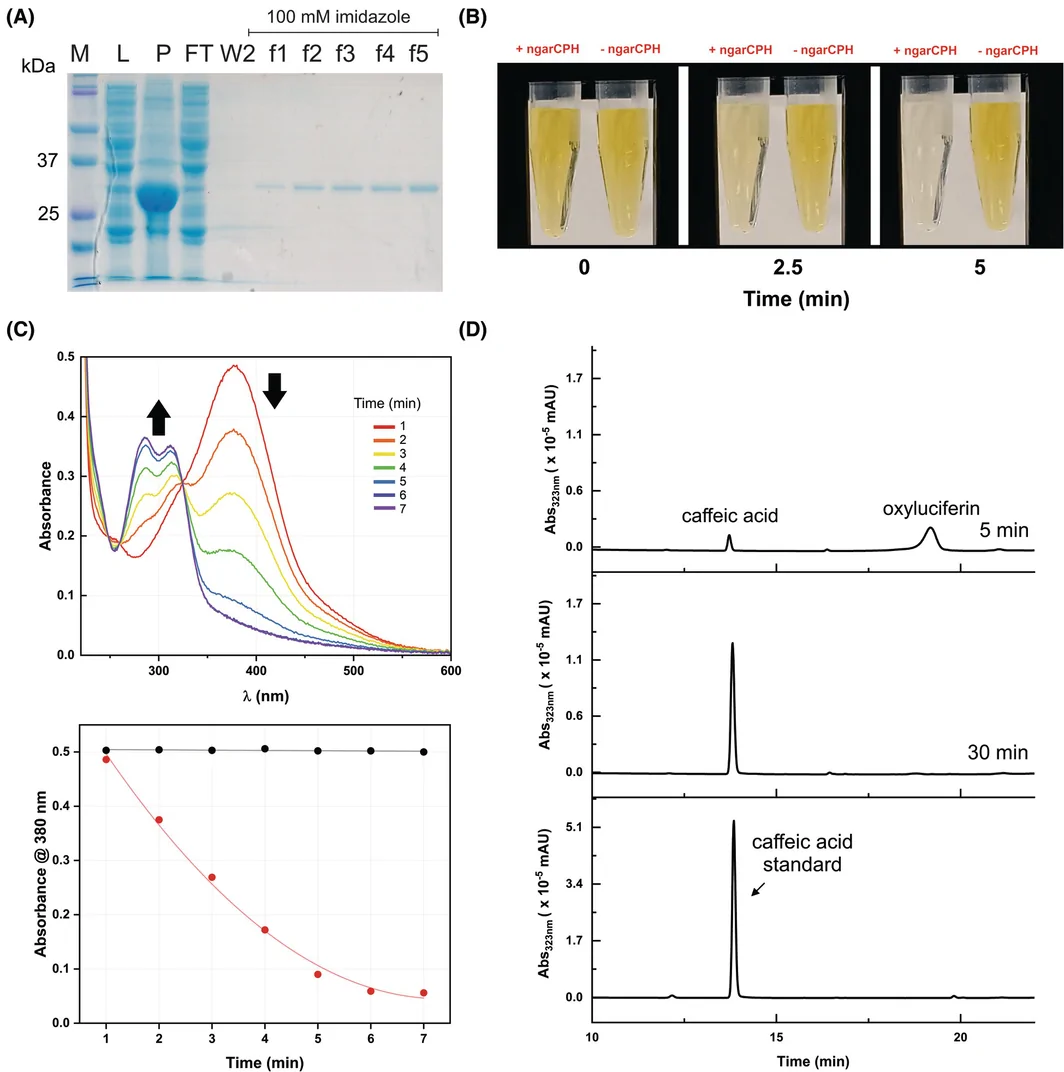
Purification and detection of oxyluciferin hydrolysis activity by the catalysis of recombinant ngarCPH. (A) SDS/PAGE showing ngarCPH purification steps: marker (M), total lysate (L), pellet (P), flow‐through (FT), wash eluate (W2), and imidazole‐eluted fractions contained the recombinant protein (f1–f5, 100 mm). (B) Visual confirmation of ngarCPH activity by color change upon reaction with oxyluciferin. (C) Time‐dependent absorbance changes at 250–600 nm (top), from 1 (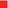), 2(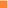), 3 (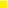), 4 (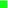), 5 (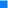), 6 (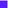) and 7 (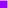) min in phosphate solution (pH 7.4). Black arrows indicate the time‐dependent appearance (upward arrow) and disappearance (downward arrow) of absorbance bands. Enzymatic activity is indicated by a decrease in absorbance at 380 nm upon addition of ngarCPH (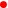, red) and control (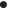, black) (bottom). (D) HPLC‐PDA analysis confirmed caffeic acid formation from the enzymatic reaction with oxyluciferin by comparison with a caffeic acid standard. The data shown are representative of two independent experiments (*n* = 2).

Since there was no described assay to evaluate the activity of CPHs, a method was developed in this work. It was found that the oxyluciferin solution exhibits an intense yellow color in phosphate buffer pH 7.4, with an absorption wavelength maximum at approximately 380 nm, which vanishes with the addition of ngarCPH (Fig. 3B) and gives rise to two new absorption bands around 300 nm, initially attributed to caffeic acid (Fig. 3C). After evaluating the entire process of oxyluciferin consumption by monitoring the total absorbance, it was identified that the wavelength of 380 nm could be selected to monitor the enzymatic activity of ngarCPH and carry out the tests to characterize its best activity conditions (Fig. 3C).

### Caffeic and pyruvic acid production by ngarCPH catalysis

Initially, the reaction products from the enzymatic catalysis of ngarCPH were evaluated by chromatography (Fig. 3D). The CPH catalyzes the hydrolytic cleavage of fungal oxyluciferin (1), yielding caffeic acid (2) and pyruvic acid (3) (Fig. 4A). Fungal oxyluciferin (1) was detected with a retention time (*R*_*t*_) of 18.0 min (Fig. 4B), confirmed by MS and UV–Vis spectrum analyses (Fig. S3). In the presence of ngarCPH, the intensity of the signal from 1 decreased, and a new signal 2 was detected from 5 min after 75 min of enzymatic reaction, being attributed to a catalytic product formed by the consumption of oxyluciferin. An increase in the signal attributed to Compound 2 was not observed in the solution without the ngarCPH enzyme. However, Compounds 4 and 5 were detected both in the absence and presence of ngarCPH, suggesting that their formation may result from nonenzymatic reactions, potentially associated with oxyluciferin degradation. The identity of Compound 2 was confirmed by comparing its MS and UV–Vis spectra with those of a caffeic acid standard solution in ethanol. Compound 2 exhibited the same retention time as the caffeic acid standard solution (*R*_*t*_ = 14.3 min) (Fig. 4B), and its MS spectrum displayed a protonated [M + H]^+^ adduct at m/z 181 (Fig. S3). The fragmentation pattern (m/z 163) indicated the expected loss of two hydroxyl groups from the catechol region, consistent with the structure of caffeic acid [11]. Despite the lower signal intensity, the *R*_*t*_, MS data, and UV–Vis spectra of Compound 2 aligned with the caffeic acid profile (Fig. S3), further confirming it as one of the products of ngarCPH catalysis.

**Fig. 4:**
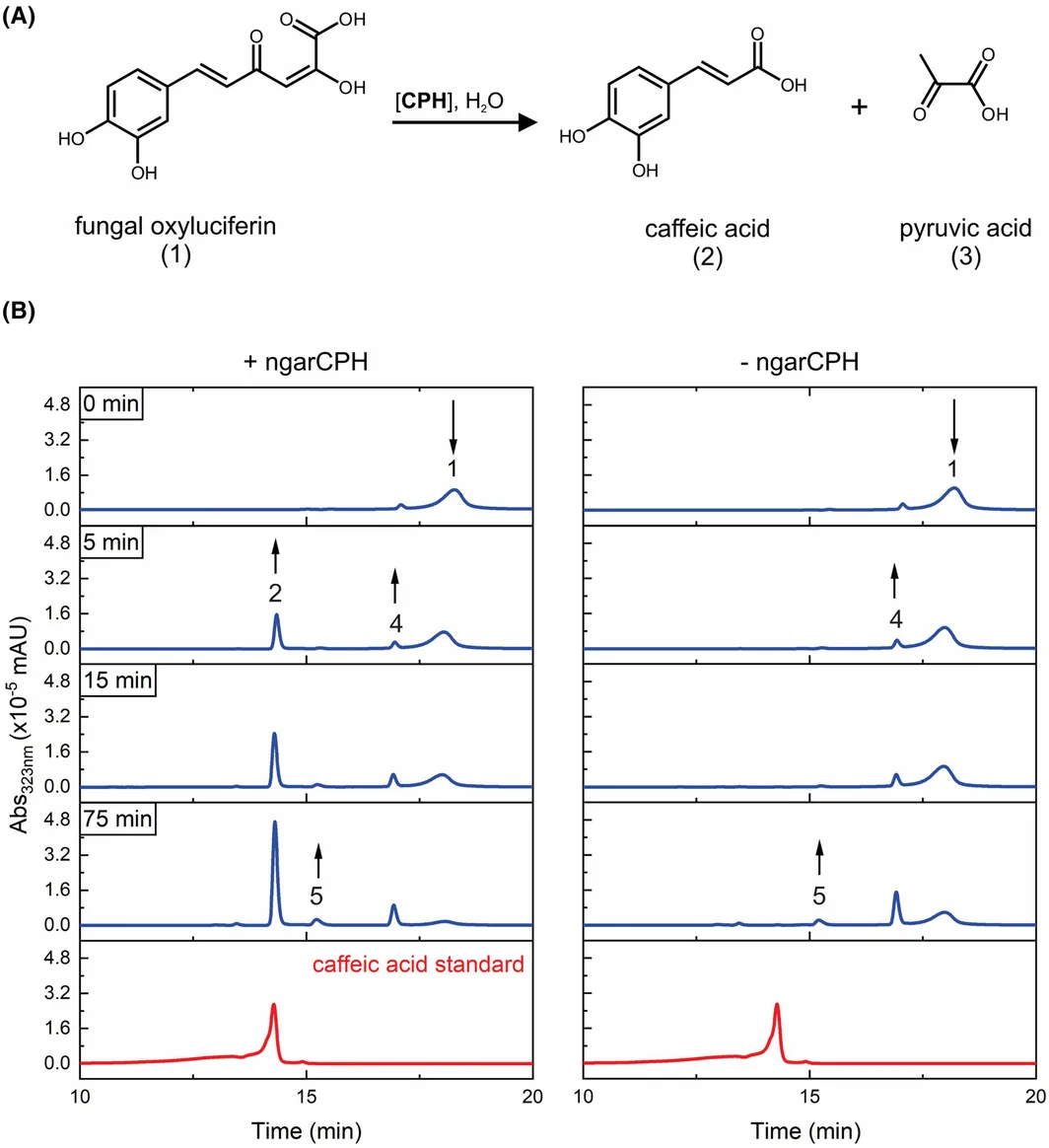
Characterization of the catalytic product of ngarCPH. (A) Hydrolysis of fungal oxyluciferin (1) into caffeic acid (2) and pyruvic acid (3) in the presence of CPH. (B, ‘+ngarCPH’) Hydrolysis of 1 by ngarCPH yields Compound 2, which has the same retention time as the caffeic acid standard solution. (B, ‘‐ ngarCPH’) The presence of unidentified Compounds 4 and 5 can be attributed to spontaneous degradation of oxyluciferin. Black arrows indicate the time‐dependent appearance (upward arrow) and disappearance (downward arrow) of HPLC peaks. Experiments were conducted at 0, 5, 15, and 75 min of enzyme‐substrate incubation. The data shown are representative of three independent experiments (*n* = 3).

Another compound expected from the hydrolysis of oxyluciferin by ngarCPH would be the pyruvic acid (3). However, it was not possible to detect it by direct chromatography with a UV–Vis detector, as was achieved for caffeic acid. To overcome this issue, a commercial enzyme kit (Sigma‐Aldrich's Pyruvate Assay Kit) was used, to which, in the presence of 3, a specific fluorescent signal can be measured (λ_ex_ = 535 nm, λ_em_ = 587 nm), allowing its detection and quantification. Using reverse‐phase HPLC‐PDA‐ESI‐MS and the Pyruvate Assay Kit, it was possible to observe the appearance of both products over time from the same reaction (Fig. 5), making it possible to quantify their concentrations by comparison with calibration curves (Fig. S4). Despite the interferences encountered during product quantification, which is addressed in the Discussion section, it was conclusively determined that the hydrolysis of oxyluciferin in the presence of ngarCPH produces both caffeic and pyruvic acids.

**Fig. 5:**
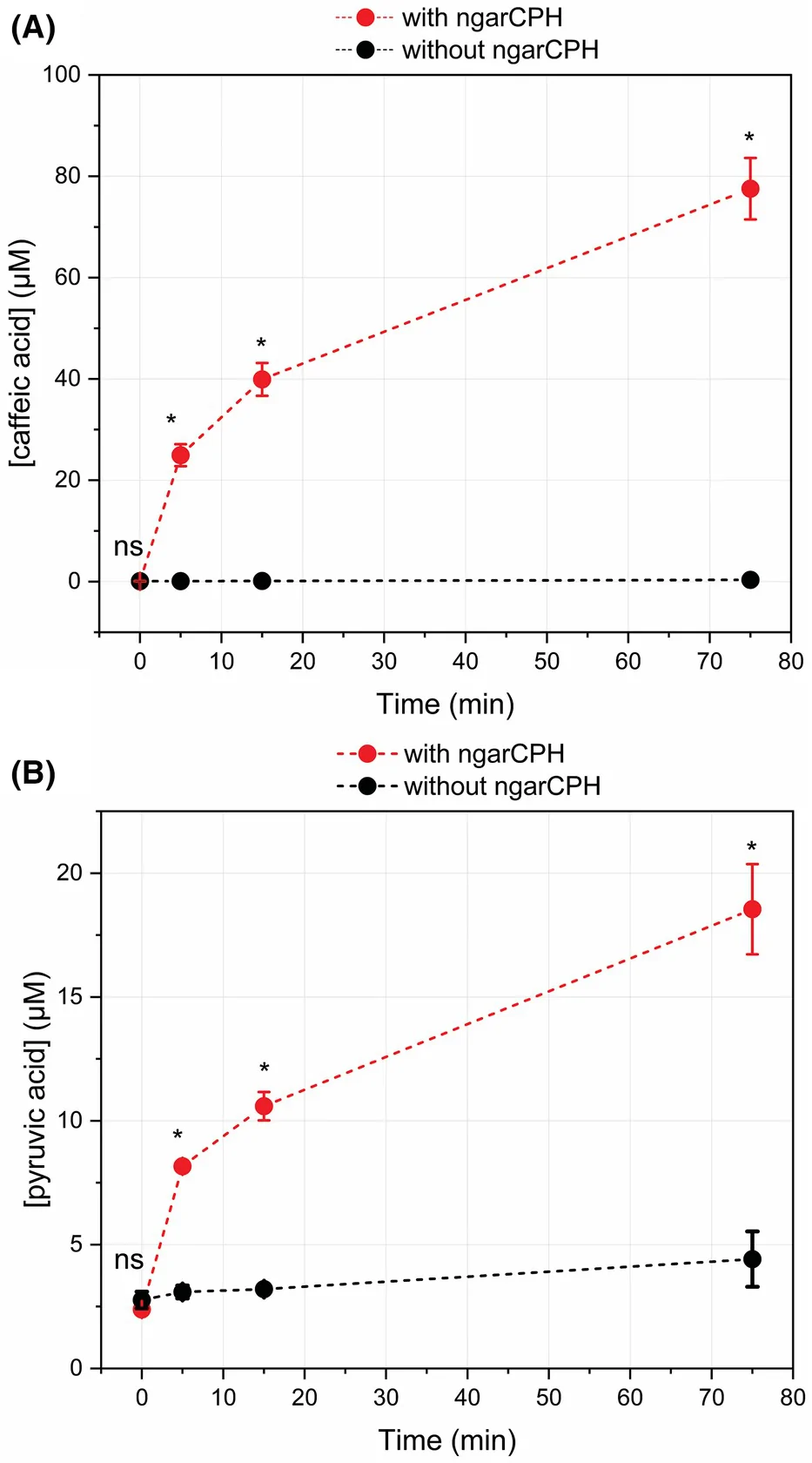
Quantification of caffeic and pyruvic acid from oxyluciferin hydrolysis by ngarCPH. Hydrolysis of oxyluciferin in the presence (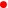, red) and absence (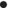, black) of ngarCPH catalysis leading to caffeic (A) and pyruvic acids (B). The samples were taken at intervals, acidified to denature the enzyme, and neutralized with a basic solution before measuring pyruvic acid. Pyruvate was detected and quantified as mentioned using the Pyruvate Assay Kit (Sigma‐Aldrich) and caffeic acid using HPLC‐PDA analysis (values are presented as mean ± SD, *n* = 3). Differences with controls (without ngarCPH) were evaluated using a two‐sample Student's *t*‐test. For (A) and (B), * *p* < 0.0001; in (A), ns *p* = 0.40; (B), ns *p* = 0.11.

### ngarCPH activity at different pH, temperature, and its steady‐state kinetic parameters

Before initiating experiments to evaluate the enzymatic activity of ngarCPH in different temperatures, it was decided to investigate whether the absorbance of the degradation products formed could interfere with the activity measurement process. To verify this, under solution conditions identical to those of the activity assays at different temperatures, the absorption in the wavelength range of 200–600 nm was measured after 8 min of incubation (Fig. S5). The results indicated a minimal change in the absorption spectrum in the absence of the enzyme, possibly due to the formation of new compounds related to the nonenzymatic degradation of oxyluciferin (Fig. 4B, ‘‐ ngarCPH’). In the presence of ngarCPH, the expected increase in absorbance between 250 and 350 nm was observed, characteristic of the formation of caffeic acid, a product of oxyluciferin hydrolysis catalysis. The nonenzymatic degradation of oxyluciferin was slow compared with the formation of caffeic acid catalyzed by ngarCPH, suggesting that it is feasible to measure the enzyme's activity at different temperatures. Based on the experiments, the optimal activity conditions for recombinant ngarCPH were determined to be within a pH range of 6.5 to 7.5 (Fig. 6A) and at a temperature of 50 °C (Fig. 6B).

**Fig. 6:**
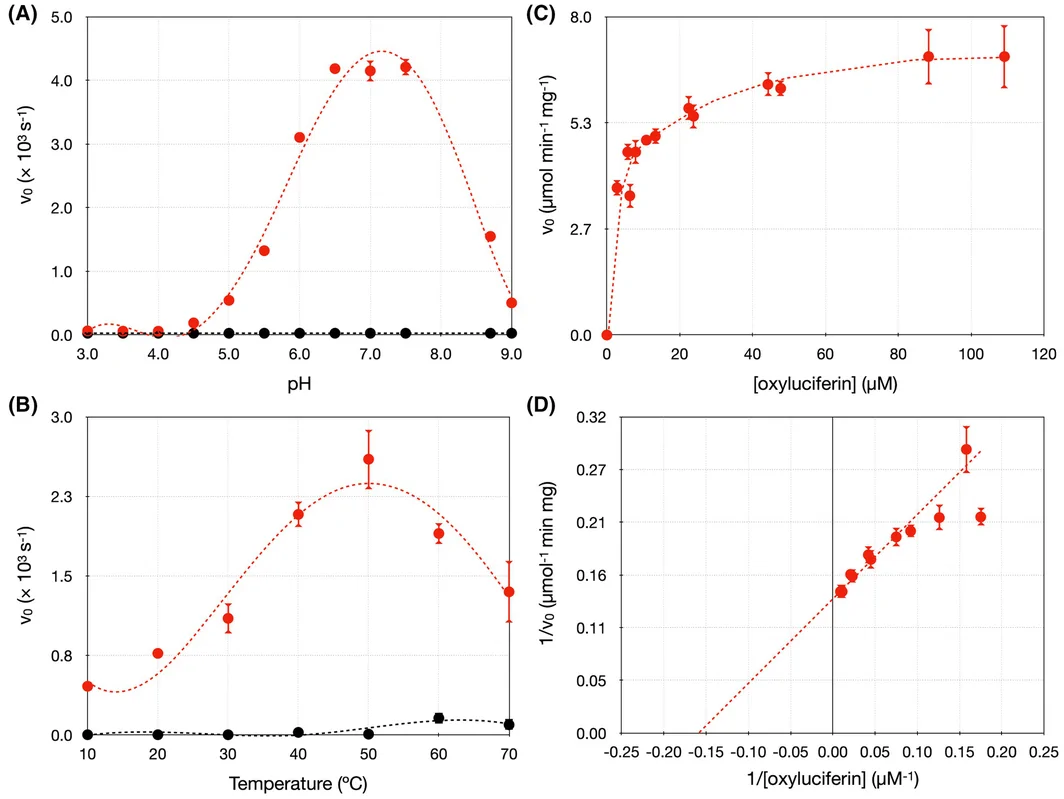
Determination of optimal conditions for ngarCPH catalysis. (A) Optimal enzymatic activity of ngarCPH at different pH (100 mm buffer solutions from pH 3 to 9, *n* = 3) and (B) temperatures in phosphate buffer (pH 7.4, *n* = 3). (C) Michaelis–Menten and (D) Lineweaver–Burk plots obtained at phosphate buffer (100 mm, pH 7.4, 12 nm ngarCPH, oxyluciferin concentration from 2.5 to 100 μm, *n* = 3). Curves show experiments performed in the presence of ngarCPH (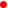, red) and in absence (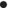, black), controls. Values are presented as mean ± SD. The red curves were fitted using Gaussian models (temperature and pH; adjusted *R* ^2^ = 0.89 and 0.98, respectively) and a linear model for the Lineweaver–Burk plot (adjusted *R* ^2^ = 0.84).

To determine the kinetic parameters, the dependence of v_0_ on the concentration of oxyluciferin was adjusted using the Michaelis–Menten equation (Fig. 6C). The concentration of oxyluciferin in solution was determined by the equation derived from the Lambert–Beer law, using the molar absorption coefficient of oxyluciferin at 380 nm established in this work (ε_380nm,oxy_ = 11 930 ± 18 m ^−1^·cm^−1^). *V*_max_ was found to be 6.4 ± 2.0 μmol·min^−1^·mg^−1^, with a *K*_*M*_ = 2.5 ± 0.4 μm, calculated from the Lineweaver–Burk plot (Fig. 6D). The constant related to the amount of substrate converted into product per unit of time (*k*_cat_ = 4 ± 1), and the catalytic efficiency (*k*_cat_/*K*_M_ = 1.4 ± 0.7 × 10^6^), which is associated with the efficiency of collisions between the enzyme and the substrate [12].

### Structural model of ngarCPH and primary sequence comparison with CPH and FAH enzymes

The ngarCPH structural conformation was predicted using the machine learning algorithm AlphaFold2 [13]. A model for the ngarCPH monomer was generated, and the structure quality metrics, such as an average of the predicted local distance difference test (pLDDT) of 97.2 and low expected positional error from predicted aligned error (PAE), indicate a high degree of reliability (Fig. S6). The spatial conformation reveals two distinct regions: an N‐terminal and a C‐terminal (Fig. 7A), which are found as characteristic of the FPH subfamily [14]. Multiple sequence alignment of deposited CPH sequences [2, 5] revealed consensus residues that are likely involved in the catalysis and metal binding in FAH enzymes [14] (Fig. 7B). Metal‐binding site prediction algorithms, Metal3D [15] and MIB2 [16], indicated a high probability of coordination of a divalent metal cation at the aligned metal‐binding residues.

**Fig. 7:**
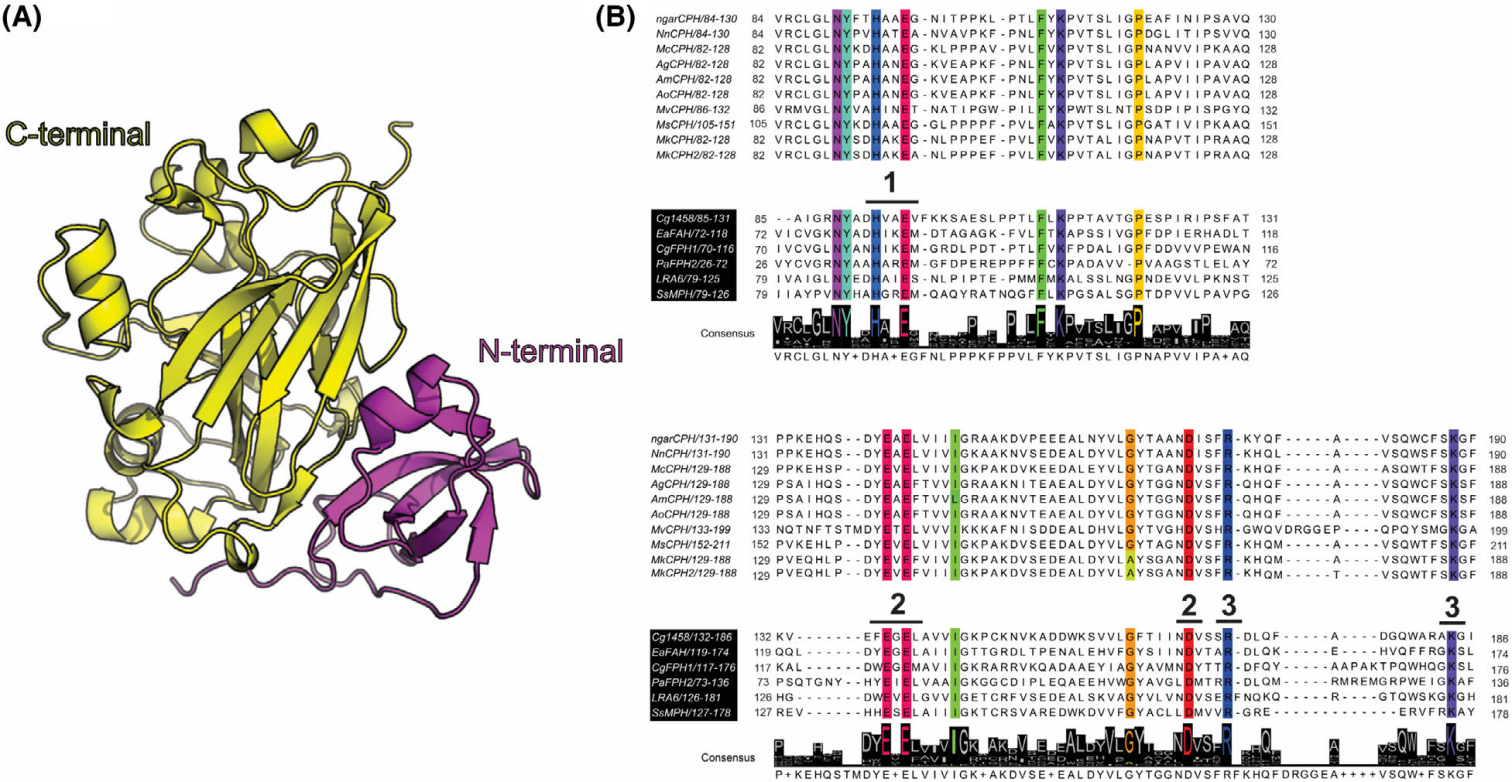
Predicted structure of the ngarCPH monomer and alignment comparison with caffeylpyruvate hydrolase (CPH) and fumarylacetoacetate hydrolase (FAH) superfamily enzymes. The model of ngarCPH highlights the N‐terminal (pink color) and C‐terminal (yellow color) regions (A). Multiple sequence alignment of CPH primary protein sequences and FAH enzymes (B), highlighting conserved motifs: (1) the catalytic Glu‐His dyad involved in water activation, (2) the Glu‐Glu‐Asp metal‐binding motif, and (3) role of the oxyanion. Highlighted colored background indicates consensus residues. The ngarCPH structural model was generated using AlphaFold2.

## Discussion

Since the discovery of the FBP, several CPH orthologs have been identified *in silico* across different bioluminescent fungi [2, 5]. The coding sequence obtained by cDNA sequencing of the *cph* gene from *N. gardneri* in this study (Table S1) differs from that previously reported, with the translated sequence described in the literature containing additional residues at Positions 6–30 and 95–105 [2]. To produce the recombinant ngarCPH, we used gene‐specific flanking primers to amplify the *cph* coding sequence, and the resulting PCR product yielded a single, unambiguous sequence. Therefore, the observed discrepancy between the sequences is likely attributable to inaccuracies in the bioinformatic prediction of the coding sequence reported in the literature [2].

Expression of recombinant ngarCPH in bacterial hosts yielded, after Ni‐NTA affinity purification, a low amount of soluble pure and enzymatically active protein, which was sufficient for the enzymatic characterization experiments. Although recombinant expression in *E. coli* cells is generally advantageous due to short production times, low cost, and high yields [17], using this system to express proteins from FBP can be challenging. In addition to ngarCPH expression, previous studies have reported that bacterial expression of *Mycena chlorophos* hispidin‐3‐hydroxylase (mcH3H) [18] and *Neonothopanus nambi luciferase* (nnLuz) [2] results in inclusion body formation and low enzymatic activity, requiring either fusion to solubility‐enhancing tags or transfer of expression to a yeast‐based system, respectively. Because these enzymes were described only recently, there is currently no information on whether they require post‐translational modification, such as glycosylation, which is known in fungi to ensure protein stability [19]. Although this study demonstrates that ngarCPH can be expressed by *E. coli*, achieving higher yields of recombinant protein may require shifting expression to a more suitable eukaryotic host.

Here, we present the first functional characterization of an enzyme classified as CPH (EC: 3.7.1.‐), for which there were no previously established activity assays. To solve this problem, a new method was developed based on monitoring absorbance during enzyme reactions. The absorption spectra of oxyluciferin, caffeic acid, and pyruvic acid between pH 3 and 9 (Fig. S7) were first examined to ensure clear spectral distinction. The solution containing oxyluciferin showed a consistent peak near 380 nm, with only a slight shift depending on the pH. Similar pH‐dependent variations in absorption have been observed for other oxyluciferins and are associated with their ionized forms [18, 20]. Caffeic acid exhibited minimal absorbance in this range, with no significant overlap. Based on these findings, 380 nm was chosen to monitor oxyluciferin consumption, enabling a reliable assay for ngarCPH activity.

Considering the FBP enzymes, it is important to evaluate the parameters of each enzyme to better understand the different steps of the process. However, due to their recent discovery, available information remains limited [2]. The enzyme ngarCPH showed maximum activity around 50 °C (Fig. 6B), notably higher than the luciferase from *N. nambi* (nnLuz), which is most active between 10 and 16 °C [2]. Although ngarCPH functions above typical fungal temperatures, its stability under these conditions is unclear, raising concerns about thermal inactivation [21, 22]. Optimal pH was between 7.0 and 7.5 (Fig. 6A), close to mcH3H [18] and slightly lower than nnLuz (pH 8.0).

These results underscore the difficulty of integrating FBP enzymes into heterologous light‐emitting systems due to differing optimal conditions. However, recent engineering of nnLuz and H3H variants improved light emission in eukaryotes [23]. For CPH, studies suggest that it can prolong light emission in plants with low levels of caffeic acid when the sequence for the *cph* gene from *N. nambi* is inserted [9]. Optimizing enzymes classified as CPH could therefore help to sustain luciferin biosynthesis. The activity assay developed here offers a valuable tool for screening CPH variants, comparing homologs and identifying efficient enzymes or inhibitors, contributing to the optimization of FBP. As this is the first functional assay for the first recombinant CPH, kinetic data remain limited for comparison.

The formation of caffeic and pyruvic acids was confirmed by a time‐dependent increase in their levels in the presence of purified ngarCPH. While caffeic acid was easily detected by HPLC‐PDA‐MS, pyruvic acid required a fluorescence‐based assay using a commercial pyruvate assay kit (Sigma‐Aldrich). The optimized protocol included enzymatic incubation, acid quenching, pH neutralization, and fluorescence detection. After 1 h, oxyluciferin was not fully consumed, with only ~80 μm caffeic acid and 20 μm pyruvate formed from ~200 μm substrate. In contrast, total oxyluciferin consumption was observed during the molar absorptivity assays. This discrepancy may be attributed to a decrease in enzymatic activity, as well as competition from oxyluciferin degradation pathways, which were evident even in the control solution during the caffeic acid quantification assays (Fig. 4). In addition, the disparity in product concentrations can also result from side reactions with the assay reagents. To assess interference, a mixture of oxyluciferin and caffeic acid (8 μm each) was tested, resulting in a 50% reduction in the pyruvate fluorescence signal, confirming assay inhibition (Fig. S8 **)**. Pyruvate detection relies on a fluorescent product generated via H₂O₂ produced by pyruvate oxidase [24]. The low pyruvate signal may stem from H_2_O_2_ consumption, as both caffeic acid and likely fungal oxyluciferin are known to scavenge reactive oxygen species, potentially disrupting fluorescent product formation [25].

Following confirmation of ngarCPH activity and product formation, the enzyme's spatial structure was investigated *in silico* using a machine learning‐predicted structure from AlphaFold2. The model showed high confidence (pLDDT scores), enabling comparison with elucidated structures from the FAH superfamily. The N‐terminal and C‐terminal regions were identified in the ngarCPH monomer, similar to enzymes of the FPH‐A subfamily [14]. The specific role of the N‐terminal region remains unclear, and the C‐terminal region is associated with the catalytic domain.

As CPH enzymes have not yet been experimentally characterized, quantum chemical calculations have suggested that catalysis of the oxyluciferin hydrolysis in three steps as follows [26]:

1. Enol‐to‐keto tautomerization via proton transfer through water molecules,
2. Base‐catalyzed nucleophilic attack by hydroxide, modeled using imidazole, mimicking a histidine residue, and
3. Carbon–carbon bond cleavage yielding caffeic acid and pyruvate.

The second step describes the formation of hydroxide by proton abstraction from water by a residue acting as a Brønsted base [26], a mechanism that has been characteristic for FAH subfamily enzymes. Comparative analysis with FAH superfamily enzymes—known for various functions, including carbon–carbon bond cleavage [27]—revealed structural similarities, particularly in active site residues and metal coordination features [14, 28]. For hydroxide formation, a Glu‐His catalytic dyad role this process and Glu‐Glu‐Asp to coordinate divalent metal ions (Mg^2+^, Mn^2+^, Zn^2+^ or Ca^2+^) [14, 29, 30] to binding and keep the stability of the substrate.

Multiple sequence alignment of primary deposited sequences encoding CPH revealed the presence of conserved catalytic residues previously reported as essential for the FAH catalytic mechanism [14], suggesting, in accordance with reaction calculation results from theoretical studies, their potential involvement in a similar mechanism. However, as this study was designed to validate the proposed activity of this novel enzyme, the *in silico* analysis should be considered a preliminary characterization. The presence and identity of the native coordinated metal remain unresolved and require experimental characterization. Moreover, additional studies are needed to confirm the roles of both individual residues and the metal cofactor.

Currently, there is no clear definition of the evolutionary advantage of CPH in bioluminescent fungi. However, based on the products generated from its enzymatic catalysis, it is hypothesized that CPH enzyme group plays a role in energy optimization, ensuring continuous bioluminescence in these species and minimizing the production of caffeic acid via the phenylalanine consumption pathway. Pyruvic acid, in eukaryotic cells, can be incorporated into mitochondria and converted into acetyl‐CoA by the pyruvate dehydrogenase complex [27]. Acetyl‐CoA, in turn, is an intermediate substrate in the tricarboxylic acid cycle and can be used to produce malonyl‐CoA by acetyl‐CoA carboxylase, an enzyme involved in fatty acid [31, 32]. This process minimizes the intracellular use of these cofactors, compensating for malonyl‐CoA consumed in the cycle for hispidin synthesis. However, it remains unclear whether the *cph* genes are fundamental or merely an evolutionary advantage acquired to minimize energy consumption in the bioluminescence metabolism of fungi. Interestingly, when considering their role in heterologous light‐emitting systems in plants, CPH enzymes could help reduce the impact on caffeic acid consumption by FBP, a compound that is also essential for lignin biosynthesis and the regulation of various physiological processes in plants [10].

Considering that the FBP shares the same enzymatic mechanism across different bioluminescent fungi [6], the confirmation of CPH activities and the assays conducted in this study significantly contribute to advancing research in other bioluminescent fungi. These results may aid in understanding the biological function of CPH, the associated metabolic regulation, and the evolutionary reasons behind the preservation of this mechanism.

## Conclusion

Fungal bioluminescence is a conserved and ancient trait observed in select species worldwide, yet its biological function remains incompletely understood [2, 5]. In this study, we provide the first functional characterization of CPH from *N. gardneri*, a key enzyme in the FBP, which hydrolytically cleaves oxyluciferin into caffeic and pyruvic acids. Using recombinant expression, purification, and a novel absorbance‐based assay, we confirmed the enzyme's catalytic role and determined its optimal activity conditions and kinetic parameters. Structural predictions using AlphaFold2, combined with molecular docking and homology modeling with FAH superfamily enzymes, revealed a conserved Glu‐His catalytic dyad and metal coordination, supporting a plausible mechanism for oxyluciferin hydrolysis.

Biochemically, the products of ngarCPH catalysis link luciferin biosynthesis with central metabolism: caffeic acid feeds back into the FBP, while pyruvic acid can enter the tricarboxylic acid cycle via acetyl‐CoA and contribute to malonyl‐CoA biosynthesis, a substrate for hispidin production. This suggests that CPH group may support energy optimization in bioluminescent fungi by recycling key metabolites and minimizing metabolic demand. The assay developed here provides a valuable tool for further studies on the role of bioluminescence under different physiological conditions, such as development, stress, and environmental stimuli [33, 34]. Additionally, confirming ngarCPH's role in the FBP opens new possibilities for its application in synthetic biology, particularly in the engineering of self‐sustained light‐emitting systems in heterologous organisms [8].

## Materials and methods

### Reagents

Sugarcane molasses (56% sucrose, Usina São José da Estiva, Novo Horizonte, SP), Luria–Bertani medium, LB (Kasvi), kanamycin sulfate (Gibco), chloramphenicol ≥ 98% (HPLC) (Sigma‐Aldrich), isopropyl thio‐β‐galactoside (IPTG) (Sigma‐Aldrich), Ni‐NTA agarose (QIAGEN), Triton‐X 100 (Nuclear), glycerol (Sigma‐Aldrich), bacteriological agar (Kasvi), yeast extract (Kasvi), NaCl PA (Synth), imidazole (Sigma‐Aldrich), phenylmethylsulfonyl fluoride (PMSF) (Thermo Scientific), β‐mercaptoethanol (Sigma‐Aldrich), caffeic acid ≥ 98% (Sigma‐Aldrich), pyruvic acid ≥ 98% (Sigma‐Aldrich), formic acid ≥ 95% (Sigma‐Aldrich), acetonitrile ≥ 99.9% (Sigma‐Aldrich), ethanol (Supelco), citric acid monohydrate PA (Vetec), glycine ≥ 99% (Sigma‐Aldrich), NaOH ≥ 98% (Sigma‐Aldrich), HCl PA (Synth), TRIS base (Ludwig Biotec), TRIzol™ Reagent (Invitrogen), chloroform ≥ 99.9% (Sigma‐Aldrich), isopropyl alcohol PA (Vetec), sodium phosphate monobasic monohydrate ≥ 98% (Sigma‐Aldrich), sodium phosphate dibasic (Sigma‐Aldrich). Oxyluciferin (≥ 99%) was kindly provided by the group of Prof. Yampolsky. Its detailed synthesis is described in literature (see supplementary material of [4]).

### *N. gardneri* mycelium cultivation

*N. gardneri* mycelium was isolated from the cultivation of fragments of basidiomes collected at the Fazenda Canabrava, Palm Forest (Altos, PI, Brazil) on Petri dishes containing a medium composed of 5% (w/v) sugarcane molasses, 1% (w/v) yeast extract, and 2% (w/v) agar. The plates were incubated at 25 °C with a relative humidity of 50–70% for approximately 15 days, and the mycelium was collected for molecular and biochemical experiments.

### Molecular cloning of *cph* coding sequence

Total RNA was isolated from freshly harvested *N. gardneri* mycelium (100 mg) using the method described by Nóbrega *et al*. (2021) [35]. Complementary DNA (cDNA) from total RNA was then synthesized using the SuperScript™ IV Reverse Transcriptase (Invitrogen), following the manufacturer's instructions. The *cph* coding sequence was amplified from the cDNA using Q5^®^ High‐Fidelity DNA Polymerase and specific primers designed to remove the stop codon and add homology ends compatible with the pET‐28b(+) plasmid. After purification, the *cph* coding sequence fragment was cloned into the pET‐28b(+) plasmid using the GeneArt™ Gibson Assembly HiFi Master Mix. The resulting plasmid, pET‐28b(+)_ngcph, was used to transform chemically competent *E. coli* DH5α cells by an adapted heat shock method [36]. Positive colonies were screened by colony PCR, and the plasmid was sequenced at Genoma USP (São Paulo, Brazil) to confirm the correct orientation of the insert.

### Recombinant expression and purification of ngarCPH

One of the plasmids was used for transformation into chemically competent Rosetta™ (DE3) strain, and the bacterial culture with the pET‐28b(+)_ngcph expression plasmid in stationary state in LB medium was stored with 10% (v/v) glycerol at −80 °C and used as a preinoculum for large‐scale protein expression.

Approximately 2 μL of the pET‐28b(+)_ngcph plasmid‐carrying expression strain stock culture was transferred to 10 mL of LB medium with 100 μg·mL^−1^ kanamycin and 34 μg·mL^−1^ chloramphenicol. The culture was allowed to develop for 16 h at 37 °C with 150 rpm agitation on a shaker. After growth, a volume of 10 mL was transferred to 1 L of LB medium with 100 μg·mL^−1^ kanamycin and 34 μg·m^−1^ chloramphenicol, maintained with agitation at 150 rpm and at 37 °C. When an optical density at 600 nm of approximately 0.7 was reached, IPTG was added to a final concentration of 0.5 mm to induce the expression of the recombinant protein in the culture bacterial medium, with the temperature maintained at 25 °C and agitation at 150 rpm. After 20 h of expression, the cells were collected by centrifugation at 4 °C, 7197 × ***g*** for 5 min and resuspended in 30 mL of Ni‐NTA lysis buffer without NaCl (50 mm phosphate, pH 7.4, 10 mm imidazole, 1 mm PMSF, 7 mm β‐mercaptoethanol). The cells were lysed under pressure using a French^®^ Pressure Cell Press (SLM^®^ Aminco) in a room set at 10 °C and for 18 disruption cycles. The cellular debris was centrifuged at 4 °C for 30 min at 7197 × ***g***, and the yellowish lysate was incubated with Ni‐NTA resin for 1 h on ice. The separation of the ngarCPH enzyme and the purification of the lysate were conducted according to the manufacturer's manual, but with two washes of 50 mL each of the respective buffer. The buffer of the first wash was composed of 50 mm phosphate, pH 7.4, 300 mm NaCl, 1 mm PMSF, 7 mm β‐mercaptoethanol, 0.5% (v/v) Triton‐X 100, and 20 mm imidazole. The second buffer had the same composition but without Triton X‐100.

For the elution and separation of contaminating proteins, a gradient of imidazole concentration was eluted through the column in 2‐mL portions, starting from 100 up to 250 mm in Ni‐NTA buffer lysis. Finally, the fractions containing only the enzyme were ultrafiltered using an Amicon^®^ Ultra 10 K‐15‐mL filter (Merck Millipore) until the volume was approximately 2 mL. The samples were aliquoted into 200 μL portions and stored at −80 °C until the start of the experiments.

Before the experiments were initiated, the solution with ngarCPH was eluted through a HiTrap™ Desalting column (Cytiva), maintaining it in a final buffer of 50 mm phosphate, 150 mm NaCl at pH 7.4. Buffer exchange was carried out on the experiment days, and protein quantification was performed using the Qubit™ Protein and Protein Broad Range (BR) Assay Kits for activity and the determination of the molar absorptivity of oxyluciferin experiments. For quantification of catalytic products assay, protein quantification was performed by measuring absorbance at 280 nm, with molar absorptivity and molecular weight of the protein (ε = 28 670 m ^−1^·cm^−1^ and MW: 33171 Da, respectively) obtained through prediction using the ProtParam tool [37].

### Development of an enzymatic assay for ngarCPH

The enzymatic assay for ngarCPH was conducted by monitoring the consumption of oxyluciferin through absorbance at a wavelength of 380 nm on NanoPhotometer^®^ NP80 (IMPLEN). As oxyluciferin is not very soluble in water, a stock solution of 50% (v/v) water:ethanol was prepared at a final concentration of 40 mm. The 50 μL aliquots were stored at −80 °C. During the experiments, the solution was thawed, kept on ice, and protected from light. For the tests with the ngarCPH enzyme, the maximum concentration of ethanol in the final solution did not exceed 0.5% (v/v). To determine ngarCPH activity, the decrease in absorbance at 380 nm (Abs_380nm_) was then monitored. During the initial minutes, Abs_380nm_ exhibited a linear decline, indicating that [S] > > [E]. This decrease was used to calculate the initial velocity (v₀) of ngarCPH activity under various pH, temperature conditions and kinetics parameters (Fig. S9).

### Detection and quantification of products from ngarCPH catalysis

A 1.5‐mL microtube containing 1 mL of 50 mm phosphate buffer at pH 7.5 and 200 μm oxyluciferin was incubated at 25 °C with agitation at 1400 rpm in the presence (+ ngarCPH, 15 nm) and absence (− ngarCPH) of the enzyme. At each specified interval (0, 5, 15, and 75 min), 200 μL samples were collected, treated with 2 μL of concentrated HCl (37%), and subsequently with 3 μL of a 6 m NaOH solution. This process aimed to denature the enzyme with acid to stop the reaction and then neutralize the pH for subsequent quantification using the Pyruvate Assay Kit (Sigma‐Aldrich). The assay was conducted using 20 μL from each reaction, following the commercial protocol. The final volume was 100 μL (5‐fold dilution). The indirect detection of pyruvic acid was performed by measuring fluorescence at 535 nm excitation and 587 nm emission after a 30‐min incubation at RT. Duplicates were prepared with independent samples.

For the remaining buffer samples, an additional 3 μL of HCl was added, and they were subsequently subjected to HPLC‐PDA‐ESI‐MS analysis by injecting 70 μL for separation and identification of caffeic acid and quantifying it against a standard curve. Triplicates were prepared with independent samples.

The conditions for reverse‐phase HPLC separation using a Luna 5 μ C18(2) 100 Å 250 × 4.6 mm column were as follows: flow rate of 0.8 mL min^−1^; solution A—ultrapure water with 0.05% (v/v) formic acid; solution B—acetonitrile with 0.05% (v/v) formic acid; 0–3 min, 10% (v/v) B, 3–18 min, 45% (v/v) B, 18–20 min, 95% (v/v) B, 20–25 min, 95% (v/v) B, 25–30 min, 10% (v/v) B. The standard curve with caffeic acid was prepared following the experiment for the identification of the molar absorptivity coefficient of oxyluciferin, with solutions at concentrations of 5, 10, 20, 60, 100, and 200 μm. Calibration curve measurements were conducted in duplicate.

### Determination of the molar absorptivity coefficient of oxyluciferin

Firstly, oxyluciferin solutions were prepared by diluting the 40 mm stock solution to obtain concentrations of 5, 10, 20, 40, and 60 μm in a 50 mm phosphate buffer pH 7.4, resulting in a final volume of 997 μL. Each solution was split into two portions of 498.5 μL each. In one of the portions, 1.5 μL of buffer (without ngarCPH) was added to measure the initial absorption at 380 nm, and in the other portion, buffer with the addition of recombinant ngarCPH at a final concentration of 25 nm was added and incubated at 28 °C until the complete consumption of oxyluciferin, *ca*. 30 min.

The caffeic acid formed in the solution by ngarCPH catalysis was quantified using a calibration curve prepared with standard solutions of caffeic acid at concentrations of 5, 10, 20, 60, and 100 μm in ethanol. A 70 μL sample was used for high‐performance liquid chromatography–photodiode array (HPLC‐PDA) analysis after enzymatic compounds separation using an Amicon^®^ Ultra 3 K‐0.5 mL column (Merck Millipore), at 3381 × ***g*** for 40 min. The conditions for reverse‐phase HPLC separation using a Luna 5 μ C18(2) 100 Å 250 × 4.6 mm column were as follows: flow rate of 0.8 mL min^−1^; solution A—ultrapure water with 0.1% (v/v) formic acid; solution B—acetonitrile with 0.1% (v/v) formic acid; 0–3 min, 10% (v/v) B, 3–18 min, 90% (v/v) B, 18–20 min, 90% (v/v) B, 20–25 min, 10% (v/v) B. The calibration curve and quantification of caffeic acid in the samples were evaluated based on the integral value of the absorbance intensity at 323 nm (Fig. S10A). Assuming that ngarCPH transformation of oxyluciferin into caffeic and pyruvic acid is equimolar [2, 4], the accurately determined concentration of caffeic acid was converted into the initial concentration of oxyluciferin, and a curve was created based on the initial absorbance at 380 nm (Fig. S10B). The molar absorptivity coefficient of oxyluciferin was determined by performing a linear regression of the Abs_380nm_ *vs*. [oxyluciferin] curve, with the slope representing the desired value. All measurements were performed in duplicate from independent samples.

### ngarCPH activity dependence on pH

Oxyluciferin was dissolved in a solution with different pH buffers to achieve a final concentration of 40 μm. In independent triplicates, ngarCPH was added to 50 nm at a final volume of 200 μL in 96‐well clear bottom plates. The buffers used included citric acid‐sodium citrate (pH 3.0–5.5), phosphate (pH 6.0–7.5), Tris–HCl (pH 8.5), and glycine‐NaOH (pH 9.0), all at a final concentration of 100 mm. Absorbance at 380 nm was recorded every 30 s for 7 min using the Infinite^®^ M200 (TECAN) at RT. The enzyme's activity was determined based on the slope value of the generated linear curve.

### ngarCPH activity dependence on temperature

In a 1.5‐mL microtube, 100 mm phosphate buffer at pH 7.4 and oxyluciferin were added to achieve a final concentration of 40 μm. The tube was placed in a Thermomixer Comfort (Eppendorf) at 10, 20, 30, 40, 50, 60, and 70 °C for 20 min, at 750 rpm, until the solution reached the desired temperature. ngarCPH was added to each microtube to achieve a final concentration of 12.5 nm, resulting in a final volume of 1 mL. The reaction was maintained under agitation at 750 rpm, and every 2 min, 200 μL from each tube was aliquoted, and 20 μL of concentrated HCl (37%) was added to denature and terminate the enzymatic reaction. Four data points were collected within an 8‐min interval. Each sample was measured for absorbance at 380 nm, and the enzyme's activity was determined as in the pH variation experiment. Experiments were conducted in triplicates from independent samples.

### Steady‐state ngarCPH kinetics

Several stock solutions of oxyluciferin were made in 50% (v/v) water:ethanol to final concentrations of 0.5, 1, 2, 4, 8, 16, 20, and 25 mm. For each test at the specific concentration, 5 μL of oxyluciferin stock solution was added to the final 1 mL of 50 mm phosphate solution pH 7.4. After addition of ngarCPH to reach the concentration of 12.5 nm, the absorbance at 380 nm at 37 °C was measured every 10 s until the first 6 absorbance values were obtained on NanoPhotometer^®^ NP80 (IMPLEN). The tests were carried out twice independently, and the *V*_max_ and *K*_*M*_ values were determined using the Lineweaver–Burke by a linear curve plot performed by using the software Origin 2024. *V*_max_ was calculated by converting μm s^−1^ into μmol min^−1^·mg^−1^ using the experimental conditions described.

### Statistical analysis

Statistical analyses were performed in OriginPro 2024, with a significance level of 0.05. Data are presented as mean ± standard error, and the number of samples is indicated in the legend of each figure. One‐way ANOVA (Tukey's *post hoc* test) was made for the interference assay using the Pyruvate Assay Kit (Fig. S8). All initial rates of oxyluciferin consumption by ngarCPH were determined by linear fitting of Abs_380nm_ *versus* time plots (OriginPro 2024), and the slope coefficients were obtained.

### Structure of ngarCPH and sequences alignment performances

With the sequencing results obtained from the coding sequence of the *cph* gene, the translation prediction was performed using the Expasy—Translate Tool server, and the primary sequence was used as a template for Alphafold 2 Collab. The bivalent metal was predicted using Mg^2+^ (87 L, 140E, 142E, 171D; score: 2.629), Ca^2+^ (140E, 142E, 171D; score: 2.796), and Mn^2+^ (140E, 142E, 171D; score: 1.621) by MIB2 [16] and Metal3D [15], (Zn) with *p* = 0.70.

The primary sequence alignment of deposited nucleotides of coding sequence (GenBank: LC435388.1) and translation of *cph* from *N. gardneri* (this work) was performed using ClustalW, with the default parameters of the MEGA11 software [38] and visualized with Jalview [39].

Multiple sequence alignment was performed using MUSCLE with the default parameters in the MEGA11 software and included the protein coding sequences download from LRA6 (PDB: 8GST) [40]; cg1458 (PDB: 4DBH) [29]; eaFAH (PDB: 6IYM) [30]; cgFPH1 (PDB: 6J5X); paFPH2 (PDB: 6J5Y); ssMPH (PDB: 6JVW) [14]; ngarCPH (sequence obtained in this study); partial protein sequences were retrieved from the supplementary material from literature [2]: nnCPH (*Neonothopanus nambi*, GenBank: BBH43519.1), agCPH (*Armillaria gallica*, GenBank: BBH43515.1), amCPH (*Armillaria mellea*, GenBank: BBH43516.1), and aoCPH (*Armillaria ostoyae*, GenBank: BBH43517.1); and translated sequences retrieved from the literature [5]: mcCPH (*Mycena chlorophos*, GenBank: KAF7299906.1); msCPH (*Mycena sanguinolenta*, GenBank: KAF7335249.1); mkCPH (*Mycena kentingensis*, GenBank: KAF7332795.1); and mvCPH (*Mycena venus*, GenBank: KAF7352220.1).

## Conflict of interest

IVY and KSS are shareholders of Light Bio, a company that commercializes applications of the fungal bioluminescence pathway.

## Author contributions

CKZ and CVS contributed to the conceptualization and illustrations. CKZ, CVS, DMMS, BBN, ZMK, EJHB, ASM, KSS, and IVY were responsible for planning and conducting the experiments. CVS was responsible for the photographs. CVS, KSS and IVY supervised the study. All authors contributed to the writing—original draft preparation and writing—review and editing, and have read and agreed to the published version of the manuscript.

## Supporting information


**Fig. S1.** RNA purification, cph amplification, and recombinant ngarCPH purification.
**Fig. S2.** Size exclusion chromatography and activity analysis of purified ngarCPH.
**Fig. S3.** MS and UV–Vis analysis of oxyluciferin and caffeic acid after ngarCPH incubation.
**Fig. S4.** Calibration curves for pyruvate and caffeic acid quantification.
**Fig. S5.** UV–Vis spectra showing temperature‐dependent oxyluciferin conversion by ngarCPH.
**Fig. S6.** Structural confidence metrics (pLDDT and PAE) for the predicted ngarCPH model.
**Fig. S7.** UV–Vis spectra of pyruvic acid, caffeic acid, and oxyluciferin at different pH values.
**Fig. S8.** Fluorescence analysis of pyruvic acid mixtures with caffeic acid or oxyluciferin.
**Fig. S9.** Kinetic analysis of oxyluciferin hydrolysis by ngarCPH as a function of temperature, pH, and substrate concentration.
**Fig. S10.** Calibration and molar absorptivity determination for caffeic acid and oxyluciferin.
**Table S1.** Sequences of ngarCPH.

## Data Availability

The datasets generated during this study are available in the Zenodo repository, https://doi.org/10.5281/zenodo.18764039. The highest‐ranking model of ngarCPH, generated using AlphaFold2, is available in the ModelArchive at https://modelarchive.org/doi/10.5452/ma‐zrzkq.
