# Supplementary material for: Caffeylpyruvate hydrolase from the bioluminescent fungus Neonothopanus gardneri is the key recycling enzyme in the fungal bioluminescence pathway
Source: FEBS J. 2026 May 19;293(20):6022–37. doi: 10.1111/febs.70554 (PMC13669639; doi:10.1111/febs.70554)
Supplement: Supplementary file 1 [file FEBS-293-6022-s001.docx]

**SUPPLEMENTARY INFORMATION**

**Caffeylpyruvate hydrolase (CPH) from the bioluminescent fungus *Neonothopanus gardneri* is the key recycling enzyme in the Fungal Bioluminescence Pathway**

Caio K Zamuner^1,2^, Douglas MM Soares^3^, Bianca B Nóbrega^1,2^, Etelvino J. H. Bechara^1,2^, Zinaida M Kaskova^4,5^, Alexander S Mishin^4,5,6^, Karen S Sarkisyan^6,7,8*^, Ilia V Yampolsky^4,5,6*^, Cassius V Stevani^1,2*^

^1^ Departamento de Química Fundamental, Instituto de Química, Universidade de São Paulo, São Paulo 05508-000, Brazil

^2^ Departamento de Bioquímica, Instituto de Química, Universidade de São Paulo, São Paulo 05508-000, Brazil

^3^ Department of Bioprocess Engineering and Biotechnology, School of Pharmaceutical Sciences, São Paulo State University, Araraquara 14800-903, Brazil

^4^ Shemyakin-Ovchinnikov Institute of Bioorganic Chemistry, Russian Academy of Sciences, 117997 Moscow, Russia

^5^ Pirogov Russian National Research Medical University, Ostrovityanova 1, Moscow 117997, Russia

^6^ Planta LLC, 121205 Moscow, Russia

^7^ Synthetic Biology Group, MRC London Institute of Medical Sciences, London, UK

^8^ Institute of Clinical Sciences, Faculty of Medicine, Imperial College London, London, UK

* Corresponding authors

**TABLES**

**Table S1.** Sequences of ngarCPH.

| **Name** | **Sequences** |
| --- | --- |
| *cph* coding sequence^a^  (897 bp) | atggcgccgattttgacaccctggactcgtctcattcgatttgtagccgttgagacgtcactcgtgcatattggtgaacccatcgaggtgactttggacgtcgggcaggcaaaatgtgaaggcaagacgatcaaagcgtacgagattattggatcagggtcggccttggacctctcagctcaggtatcgaagaatgtgctaaccgtaaaggaactcttgatgccgctttcgagagaagaggtcaagactgtgcggtgcttgggactgaactattttactcatgctgccgaaggtaacatcactcctccaaaattaccgactttgttctataagccagtgacttcactcatcggacctgaggcgttcattaatattccttccgctgttcaaccaccgaaggagcatcagtccgattatgaagcggagttggtaattattattgggagagcggcgaaggatgtaccggaagaggaggctttgaattatgttttgggatacaccgccgccaacgacatttcatttaggaaatatcaatttgcagtttcccagtggtgtttttcgaaaggtttcgataatacaaacccaatcggtccgtgcatcgtttccgcatcttccattccgaacccgcaagacatccagatccaatgcaaactgaacgggaatgtcgttcagaatggaaacaccagtgatcaaatttttaatatcaagaaaacagtcgcttttttgtcgcaaggaacaacacttgagtcaggatcaatcatcctgaccggtacgcctggcggagtgggatttgtgcgcgatccaccgctttaccttaaagatggagatgaagtagtgacttggattggaagtggggttggaagtttagtcaacgtagtaaaagaagagaagactctt |
| ngarCPH recombinant protein  (305 aa) | MAPILTPWTRLIRFVAVETSLVHIGEPIEVTLDVGQAKCEGKTIKAYEIIGSGSALDLSAQVSKNVLTVKELLMPLSREEVKTVRCLGLNYFTHAAEGNITPPKLPTLFYKPVTSLIGPEAFINIPSAVQPPKEHQSDYEAELVIIIGRAAKDVPEEEALNYVLGYTAANDISFRKYQFAVSQWCFSKGFDNTNPIGPCIVSASSIPNPQDIQIQCKLNGNVVQNGNTSDQIFNIKKTVAFLSQGTTLESGSIILTGTPGGVGFVRDPPLYLKDGDEVVTWIGSGVGSLVNVVKEEKTLHHHHHH |

In red the modification for the addition of the histidine tail for purification by affinity chromatography.

^a^ The stop codons are not described.

**SUPPLEMENTARY FIGURES**

**Figure S1.** RNA total purified from *N. gardneri* and the c*ph* coding sequence amplified by PCR using cDNA template synthesized from RNA total (A), and purified ~33kDa ngarCPH recombinant from *E. coli* Rosetta after Ni-NTA resin elution with imidazole gradient (B). The data shown are representative of two independent experiments (n = 2).

k_obs_ (s^-1^)

**Figure S2.** Size exclusion chromatography with the concentrated fraction of purified enzyme. An amount of approximately 0.9 mg was injected into a Hiprep 16/60 Sephacryl S-220R column (GE Healthcare) (Cytiva), with a flow rate of 0.5 mL/min of 50 mM phosphate buffer, pH 7.4, and 150 mM NaCl (A); Fractions 1-9 were collected and confirmed for oxyluciferin hydrolysis activity, which was measured by k_obs_ (B); visualization of the proteins in the fractions with activity by SDS-PAGE (C). The experiment was performed once.

**Figure S3.** Analysis of separation compounds after ngarCPH incubation. MS (A) and UV-Vis (B) of oxyluciferin, product of the catalysis and caffeic acid standard.

**Figure S4.** Calibration curve for pyruvate (A) following the Pyruvate Assay Kit procedure, and calibration curve for caffeic acid (B) using HPLC. Error bars represent standard deviation (values are presented as mean ± SD, n=2). The calibration curves for pyruvate (Pyruvate Assay Kit, adjusted R² = 0.998) and caffeic acid (HPLC, adjusted R² = 0.999) were fitted using a simple linear model (OriginPro 2024).

+ngarCPH	-ngarCPH

**Figure S5.** Absorbance UV-Vis spectrum of 40 µM oxyluciferin with (+ngarCPH) or without (-ngarCPH) at different temperatures in 100 mM phosphate buffer, pH 7.4. The formation of caffeic is detected by appearance between 300 nm absorbance spectrum only in presence of the ngarCPH after 8 min of the incubation. The data shown are representative of two independent experiments (n = 2).

**Figure S6.** pLDDT values (A) and PAE score (B) from the best ranked monomer model of ngarCPH, generated using AlphaFold2.

**Figure S7.** Absorbance UV-Vis spectrum of pyruvic acid, caffeic acid and oxyluciferin at different pH. All components are in concentration of 40 µM in 100 mM citrate-acid citric buffer (pH 3.0, 4.0, 5.0 and 6.1); 100 mM phosphate buffer (pH 7.0 and 8.1); and, 100 mM glycine-NaOH buffer (pH 9.0). The data shown are representative of three independent experiments (n = 3).

**Figure S8.** Evaluation of fluorescence in mixtures containing pyruvic acid. The combination of pyruvic acid with caffeic acid or oxyluciferin, at equimolar concentrations (50 µM), results in a significant reduction in fluorescence emission compared to the condition containing only pyruvic acid (values are presented as mean ± SD, n = 2). One-way ANOVA (Tukey’s post hoc test) with 0.05 significance statistic (OriginPro 2024).

**Figure S9.** Kinetic curves obtained from the reaction of oxyluciferin (40 µM) with ngarCPH (50 nM) in phosphate buffer 100 mM pH 7.4 at different temperatures (A), ngarCPH (12.5 nM) at different pH (B, at 22ºC) and the enzymatic reaction at different oxyluciferin concentrations (C, pH7.4 and 37ºC). Values are presented as mean ± SD, n = 3.

**Figure S10.** Calibration curve obtained by integration of the absorbance peak area at 323 nm (Abs₃₂₃_nm_) versus the concentration of the caffeic acid standard, used to quantify caffeic acid produced by ngarCPH-catalyzed enzymatic reactions (A). From the determined concentration of oxyluciferin, a calibration curve was made with the absorbance at 380nm (Abs_380nm_) to determine the molar absorptivity coefficient (B). Error bars represent standard deviation (values are presented as mean ± SD, n = 2). The linear calibration curves for caffeic acid (adjusted R² = 0.999) and oxyluciferin (adjusted R² = 0.999) were fitted using a simple linear model (OriginPro 2024).
